# Serum concentration of asprosin in new-onset type 2 diabetes

**DOI:** 10.1186/s13098-020-00564-w

**Published:** 2020-07-23

**Authors:** Shakiba Naiemian, Mohsen Naeemipour, Mehdi Zarei, Moslem Lari Najafi, Ali Gohari, Mohammad Reza Behroozikhah, Hafez Heydari, Mohammad Miri

**Affiliations:** 1 Department of Bitechnology, Sabzevar Branch, Islamic Azad University of Sabzevar, Sabzevar, Iran; 2 Cellular and Molecular Research Center, Sabzevar University of Medical Sciences, Sabzevar, Iran; 3 Department of Physical Education and Sport Science, Faculty of Human Science, University of Neyshabour, Neyshabour, Iran; 4 Pharmaceutical Sciences and Cosmetic Products Research Center, Kerman University of Medical Sciences, Kerman, Iran; 5 Non-Communicable Diseases Research Center, Department of Environmental Health, School of Public Health, Sabzevar University of Medical Sciences, Sabzevar, Iran

**Keywords:** Diabetes mellitus, Asprosin, Adipokine, Insulin resistance

## Abstract

**Background:**

Asprosin, a newly identified adipokine, is pathologically increased in individuals with insulin resistance. However, the available evidence on the association of asprosin and type 2 diabetes mellitus (T2DM) status is still scarce. Therefore, this study aimed to determine the relationship between serum concentrations of asprosin and T2DM status.

**Methods:**

This observational study was performed based on 194 adults (97 newly diagnosed T2DM and 97 healthy individuals). Anthropometric and biochemical variables were determined in all participants. Serum concentrations of asprosin were measured using enzyme-linked immunosorbent assay (ELISA).

**Results:**

In patients with T2DM, the serum concentrations of asprosin were significantly higher than the healthy controls (4.18 [IQR: 4.4] vs. 3.5 [IQR: 1.85], P < 0.001). The concentrations of asprosin were significantly correlated with body mass index (BMI) and fasting blood glucose (FBG) in healthy subjects and with BMI, FBG, hemoglobin A1c (HbA1c), homeostatic model assessment of insulin resistance (HOMA-IR), and quantitative insulin check index (QUICKI), triacylglycerol (TAG) and total cholesterol/high-density lipoprotein cholesterol (TC/HDL-C) ratio in the T2DM group. In fully adjusted model, the odds ratio (OR) of T2DM with serum concentrations of asprosin was approximately 1.547 (95% CI 1.293–1.850, P < 0.001) compared to the control group. Multiple stepwise regression analysis indicated that FBG and HOMA-IR were independently associated with asprosin in T2DM.

**Conclusion:**

Our findings indicated that serum concentrations of asprosin are increased in patients with T2DM. Also, asprosin is correlated with insulin resistance and TC/HDL-C ratio (atherosclerotic risk factor of cardiovascular diseases) in patients with T2DM.

## Background

Obesity is a strong risk factor of type 2 diabetes mellitus (T2DM) [1]. The global growth of obesity largely explains the reason behind the dramatic increase in the incidence and prevalence of T2DM over the past 20 years [2]. However, the mechanisms that link these conditions are not fully understood [3]. Improving our knowledge about underlying mechanisms can lead to the identification and development of new treatment options.

Adipokines are cytokines secreted from adipose tissue, which affect a broad spectrum of biological processes [4–7]. Some of these adipokines, such as resistin and leptin, antagonize insulin function in peripheral tissues, especially in the liver and skeletal muscles, which leads to insulin resistance [7–10]. In contrast, adiponectin increases insulin sensitivity [11, 12]. The balance between adipokines is thought to play an important role in regulating insulin sensitivity [13]. Asprosin, a new adipokine, was identified in 2016 by Romere.et al. [14]. This adipokine is produced in response to starvation in adipocytes due to the breakdown of the C-terminal domain of profibrillin [14]. Asprosin rapidly releases glucose from liver cells by activating the G protein-cAMP-PKA pathway [14]. The olfactory receptor (OLFR734) acts as a receptor for asprosin in hepatocytes and is involved in hepatic glucose production [15]. Olfr734 knockout mice have been shown a poor response to asprosin, low cAMP and glucose production, and increased insulin sensitivity [16]. Some studies have shown that serum concentration of asprosin is associated with insulin sensitivity [15, 17–20]. Injection of recombinant asprosin increases glucose and causes hyperinsulinemia, while injection of anti-asprosin antibodies improves glucose tolerance and insulin resistance [14]. Elevated serum asprosin concentrations are pathophysiologically in patients with metabolic syndrome [14]. The association between asprosin and T2DM has been investigated in some studies [19–21]; however, for two major reasons, more evidence is needed to confirm the role of asprosin in the development of T2DM. Firstly, ethnic variation usually affects adipokines concentrations. The results of studies carried out in different populations suggest that inconsistent results may be obtained when adipokines are examined in different populations [22–25]. Secondly, some of the contradictory results are observed regarding the correlation of asprosin with lipid metabolism in previous studies [15, 20, 21].

Therefore, this observational study was designed to compare the serum concentration of asprosin in new-onset T2DM patients with healthy subjects.

## Materials and methods

### Subjects

All participants of this observational study signed a consent form approved by the Ethics Committee of Sabzevar University of Medical Sciences (Code of Ethics: IR.MEDSAB.REC.1398.39) before including to study. From August to December 2018, 97 newly diagnosed patients with T2DM (50 male and 47 female) were recruited from the Diabetes Center of Vasei Hospital (Sabzevar, Iran). Over the same period, 97 healthy subjects with normal glucose tolerance (NGT) (50 male and 47 female) were selected as healthy controls in consultation with a specialist physician. Inclusion criteria for the T2DM group were: having T2DM, no treatment for diabetes, including drugs that affect glucose tolerance and insulin release, dietary control, exercise therapy. Exclusion criteria were: obesity (BMI > 30 kg/m^2^), cancer, thyroid disorders, liver disease, infection, inflammation, pregnancy, hypertension, cardiovascular diseases, alcohol consumption, and smoking. Demographic information and lifestyle data were collected through a pre-designed questionnaire by face to face interview.

### Diagnostic criteria

NGT and T2DM were diagnosed in 2014, according to American Diabetes Association’s (ADA) recommended criteria [26]. Subjects with fasting blood glucose (FBG) ≥ 126 mg/dL, or hemoglobin A1c (HbA1c) ≥ 6.5%, or oral glucose tolerance test (OGTT) 2 h post-load plasma glucose ≥ 200 mg/dL were considered as T2DM patients. Subjects with FBG < 110 mg/dL, HbA1c < 5.7% and OGTT-2 h post-loading plasma glucose < 140 mg/dL were considered as the control group. Hypertension was defined as systolic blood pressure ≥ 140 mmHg or a diastolic blood pressure ≥ 90 mmHg.

### Anthropometric data collection

Body weight and height were measured by a trained nurse. Body mass index (BMI) (kg/m^2^) was calculated by dividing weight (kg) by height in squared meters (m^2^). Before each person’s blood pressure was measured, they rested for at least 10 min. Blood pressure was measured three times, and the mean of these values was considered as blood pressure.

### Biochemical measurements

Blood samples were collected after at least 12 h overnight fasting between 07:30 -08:30 a.m. Blood samples were transferred to the laboratory in serum separator tubes with clot activator in less than 2 h. Glucose, triacylglycerol (TAG), total cholesterol (TC) were measured using enzymatic assays (Pars Azmoon, Tehran, Iran), and high-density lipoprotein cholesterol (HDL-C) concentration was measured using a direct method (Pars Azmoon, Tehran, Iran). Low-density lipoprotein cholesterol (LDL-C) concentration was calculated using the Friedewald formula [27]. Insulin was assessed using enzyme-linked immunosorbent assay (ELISA) kit (monobind Inc., USA). HbA1c concentration was measured using standard methods by PishtazTeb kit. Sera were stored at − 80 °C until subsequent analysis. The homeostatic model assessment of insulin resistance (HOMA-IR), HOMA-β, HOMA-S and quantitative insulin check index (QUICKI) were used as insulin resistance and insulin sensitivity indices, respectively. These indices were calculated using HOMA2-calculator (available at https://www.dtu.ox.ac.uk/homacalculator/) and QUICKI calculator online software (available at https://www.mdapp.co/insulin-sensitivity-quicki-calculator-324/).

### Assessment of serum asprosin concentrations

Serum asprosin concentrations were determined by a commercial enzyme-linked immunosorbent (ELISA) kit according to the manufactures instructions (Cat. No: CK-E91570; EASTBIOPHARM, China). The intra-assay coefficient of variation was < 10%, and the interassay coefficient of variation was < 12%.

### Statistical analysis

All statistical analyses were conducted by SPSS software version 23.0 (IBM, Armonk, NY). The distribution of the data was tested by the Shapiro–Wilk test. Mann–Whitney and *t* test were used to compare the continuous difference variables between groups. A Chi squared test was applied for comparing categorical variables between groups. The participants were divided into three groups based on asprosin concentration with cutoff 3 ng/mL, and analysis of variance (ANOVA) was performed for group comparison. Correlation between different variables and asprosin concentrations was tested using Spearman correlation coefficient. Multivariate logistic regression analysis was used to analyze the association between serum concentrations of asprosin and T2DM. Moreover, linear stepwise regression model has applied to develop a model based on potential predictor variables. P values < 0.05 were regarded as statistically significant.

## Results

### General characteristics of individuals

**Fig. 1:**
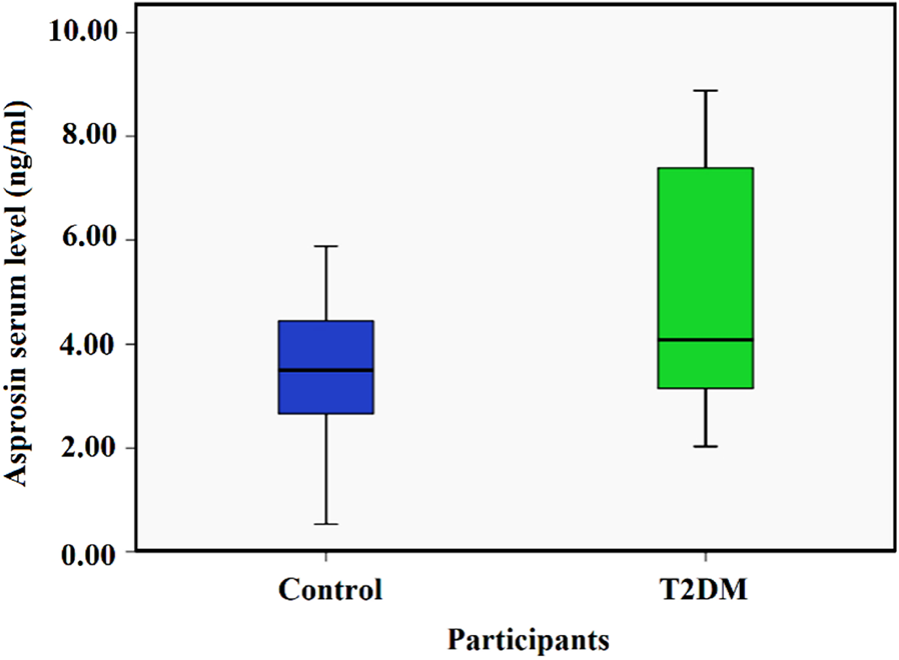
Serum concentration of asprosin in control and T2DM groups. Data shows there is a significant increase in the serum concentrations of asprosin in T2DM patients in comparison to the control group (3.50 [IQR 1.85] vs. 4.18 [IQR 4.4], P value < 0.001)

The clinical parameters of the 194 participants are shown in Table 1. FBG, HbA1c, TAG, LDL-C and fasting insulin concentrations, LDL-C/HDL-C and TC/HDL-C ratio and HOMA-IR were significantly higher in patients with T2DM than in the control group. However, HDL-C concentration, HOMA-β, HOMA-S and QUICKI were significantly lower in patients with T2DM compared to the control group. There was no significant difference between TC in the two groups (Table 1). Moreover, the asprosin concentration was not significantly different between men and women within each group and between patients with T2DM and control group (*P *> 0.05). The serum concentration of asprosin was significantly higher in T2DM patients compared to healthy controls (Fig. 1).

**Table 1:** Clinical characteristics of 194 participants in this study. There is a significant increase in parameters related to T2DM which confirms the state of disease and health of control group

Parameter	Control	T2DM	*P*-*value*
N	97	97	–
Sex (M/F)	50/47	50/47	–
Age (year)^#^	52 (10)	54 (7)	0.290
BMI (kg/m^2^)^#^	26.66 (3.01)	27 (3.27)	0.272
FBG (mg/dL)^#^	88.5 (15.0)	150 (69)	< 0.001
HbA1c (%)^#^	5.2 (0.6)	7.3 (1.92)	< 0.001
Insulin (mIU/L)^#^	5.35 (4.13)	11.77 (5.3)	< 0.001
HOMA-IR^#^	0.68 (0.51)	1.79 (0.75)	< 0.001
HOMA-β^#^	80.55 (39.15)	38.90 (41.20)	< 0.001
HOMA-S^#^	145.9 (108.02)	55.10 (24.50)	< 0.001
QUICKI^#^	0.38 (0.04)	0.30 (.03)	< 0.001
TC (mg/dL)*	197.21 ± 39.1	193.75 ± 43.51	0.72
TAG (mg/dL)^#^	137.50 (66)	182 (151)	< 0.001
HDL-C (mg/dL)^#^	54 (16)	40 (8)	< 0.001
LDL-C (mg/dL)^#^	96 (27)	143 (43)	< 0.001
LDL-C/HDL-C^#^	1.96 (0.54)	3.57 (1.00)	< 0.001
TC/HDL-C^#^	3.75 (0.87)	4.84 (1.39)	< 0.001
TAG/HDL-C^#^	3.56 (1.72)	3.88 (2.45)	0.626
Asprosin (ng/mL)*	3.50 (1.85)	4.18 (4.4)	< 0.001

*BMI* body mass index; *HOMA-IR* homeostatic model assessment of insulin resistance, *QUICKI* quantitative insulin check index

* Data normally distributed are shown as mean ± SD. Independent sample t test was perofrmed

^#^Data with skewed distribution are shown as median (IQR). Mann–Whitney U test was performed

### Asprosin concentration and clinical parameters

**Fig. 2:**
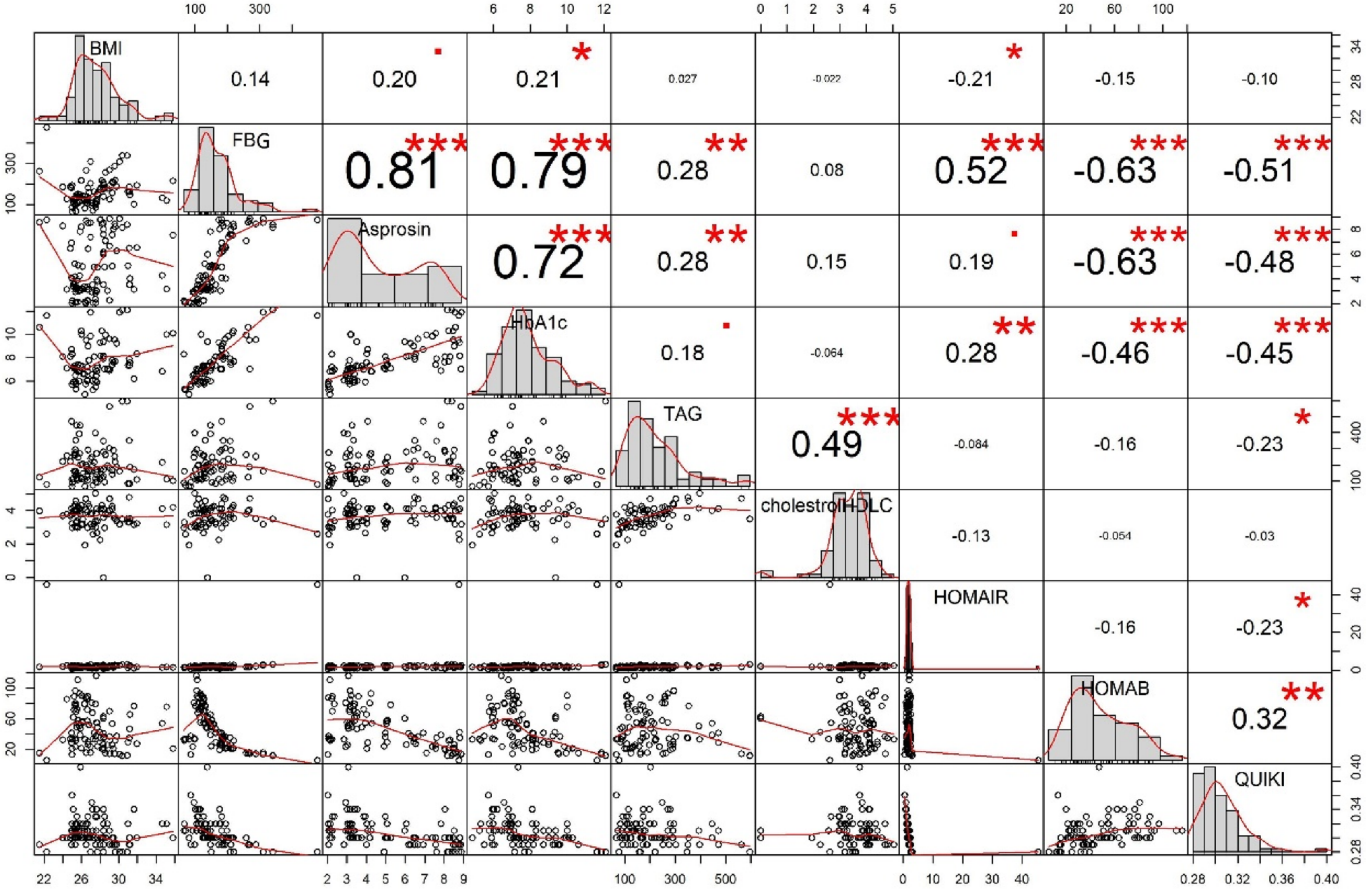
Correlation matrix, scatter plot, and histogram of BMI, FBG, HbA1c, HOMA-IR, HOMA-β, and QUICKI, TAG and TC/HDL-C ratio in the T2DM group

Spearman correlation analysis showed that in the control group, serum concentrations of asprosin were significantly correlated with BMI (*r* = 0.454, *P *< 0.001) and FBG (*r* = 0.720, *P *< 0.001) (Table 2). In the T2DM group, asprosin was positively correlated with BMI (*r* = 0.285, *P *< 0.01), FBG (*r* = 0.875, *P *< 0.001), HbA1c (*r* = 0.746, *P *< 0.001), HOMA-IR (*r* = 0.214, *P *= 0.039), TAG (*r* = 0.254, *P *= 0.015) and TC/HDL-C (*r* = 0.214, *P *= 0.044). Moreover, asprosin was negatively correlated with HOMA-β (*r* = − 0.567, *P *< 0.001) and QUICKI (*r* = − 0.522, *P *< 0.001) in the T2DM group (Table 2 and Fig. 2).

**Table 2:** Spearman correlation analysis between possible affecting factors and asprosin concentrations in control and T2DM groups

Parameter	Control		T2DM	
	r	p	r	p
Age (year)	0.139	0.175	− 0.116	0.269
BMI (mg/k^2^)	0.454**	< 0.001	0.285**	0.006
FBG (mg/dL)	0.720**	< 0.001	0.875**	< 0.001
HbA1c (%)	− 0.076	0.462	0.746**	< 0.001
Insulin (mIU/L)	− 0.139	0.174	− 0.079	0.454
HOMA-IR	− 0.082	0.422	0.214*	0.039
HOMA-β	− 0.574**	< 0.001	− 0.567**	< 0.001
HOMA-S	0.051	0.621	− 0.135	0.198
QUICKI	− 0.117	0.255	− 0.522**	< 0.001
TC (mg/dL)	0.041	0.691	0.142	0.178
TAG (mg/dL)	0.036	0.729	0.254*	0.015
HDL-C (mg/dL)	0.121	0.239	0.060	0.580
LDL-C (mg/dL)	0.139	0.175	0.074	0.488
LDL-C/HDL-C	0.064	0.537	0.065	0.544
TC/HDL-C	− 0.059	0.564	0.214*	0.044
TAG/HDL-C	0.030	0.774	0.205	0.054

Our analysis showed BMI, FBG and, HOMA-β were significantly correlated with the asprosin concentration in both groups. We also found that only in T2DM patient HbA1C, HOMA-IR, QUICKI, TAG and TC/HDL-C are in correlation with asprosin in serum

*BMI* body mass index, *FBG* fasting blood sugar, *HbA1c* hemoglobin A1c, *TC* total cholesterol, *TAG* triacylglycerol, *HDL-C* HDL cholesterol, *LDL-C* LDL cholesterol, *HOMA-IR* homeostatic model assessment of insulin resistance, HOMA-β, *QUICKI* quantitative insulin sensitivity check index and triacylglycerol (TAG) and total cholesterol/HDL cholesterol (TC/HDL-C) ratio

The results of the stepwise regression model are presented in Table 3. Results of possible clinical parameters (BMI, HOMA-β, HOMA-S, QUICKI, HbA1c, TAG, TC, insulin, LDL-C, HDL-C, LDL-C/HDL-C, TC/HDL-C, and TAG/HDL-C) effect on asprosin indicated that FBG and HOMA-IR independently associated with serum concentrations of asprosin in T2DM.

**Table 3:** Multiple stepwise regression analysis: independent factors associated with serum asprosin concentrations

Independent factors	β (unstandardized coefficient)	Std. error	t	P-value	95.0% confidence interval for β	
					Lower Bound	Upper Bound
Constant	0.206	0.034	6.038	< 0.001	0.138	0.274
FBG (mg/dL)	0.003	0.000	13.738	<0.001	0.002	0.003
HOMA-IR	− 0.015	0.003	− 4.703	<0.001	− 0.021	− 0.008

*FBG* fasting blood sugar, *HOMA-IR* homeostatic model assessment of insulin resistance

### Serum concentrations of asprosin and T2DM

**Fig. 3:**
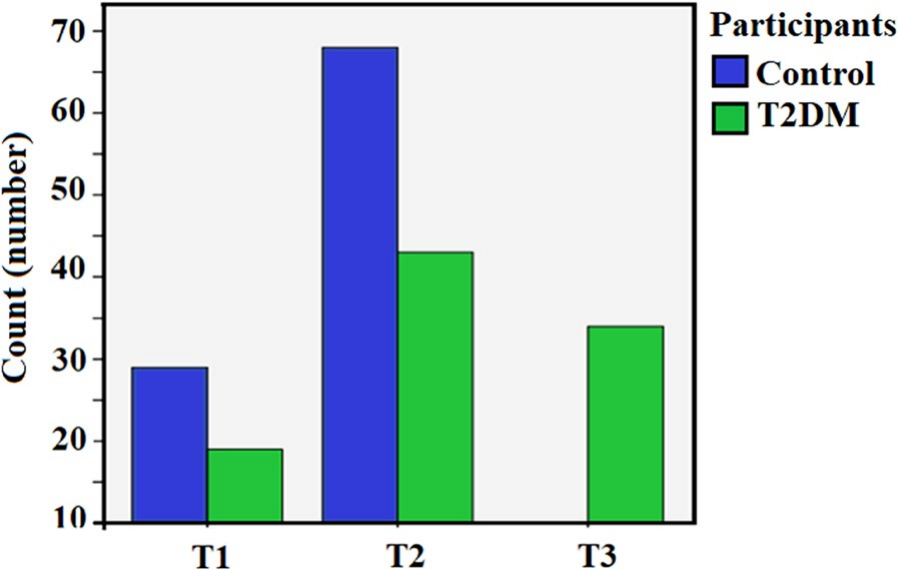
Number of individuals present in each tertile. Most of our study population was situated in T1 and T2s. However, the number of T2DM patients was higher and T2 and T3 in comparison to T1. Most of the control was placed in T2. The most interesting result is that there are only T2DM patients on the T3

All subjects were divided into tertiles based on serum concentrations of asprosin (T1: < 3 ng/mL, T2: 3–6 ng/mL and T3: > 6 ng/mL). The clinical parameters for each category are listed in Table 4. Parameters such as BMI, FBG, HbA1c, fasting insulin, TAG, HDL-C, TAG/HDL-C, and HOMA-IR increased in correspondence to tertiles. Parameters such as HOMA-β, HOMA-S and QUICKI decreased in correspondence to tertiles. The trend Chi square test showed that the number of T2DM patients increased with elevation of asprosin concentrations among the tertiles (Fig. 3). Only patients with T2DM were placed in the third tertile.

**Table 4:** Distribution of clinical parameters of participants in different tertiles based on serum concentrations of asprosin in all subjects

Variable	T1	T2	T3	P-value
Asprosin (ng/mL)	2.18 (0.4)	3.65 (1.58)	8.04 (1.74)	< 0.001
Age (year)	53 (13)	52 (8)	53 (8)	0.822
BMI (kg/m^2^)	25.79 (1.82)	26.75 (3)	28.38 (2.98)	< 0.001
FBG (mg/dL)	83.5 (15)	100 (36)	209 (83)	< 0.001
Insulin (mIU/L)	7.7 (5.01)	7.5 (5.45)	10.55 (4.18)	< 0.001
HbA1c (%)	5.50 (0.7)	5.73 (1.72)	8.99 (2.09)	< 0.001
TC (mg/dL)	198.5 (61)	194 (63)	197 (41)	0.403
TAG (mg/dL)	136 (87)	153 (79)	223 (145)	< 0.001
HDL-C (mg/dL)	42.5 (18)	43 (15)	52 (17)	< 0.001
LDL-C (mg/dL)	124.5 (49)	125 (56)	96 (21)	0.002
LDL-C/HDL-C	3.11 (1.69)	2.85 (1.74)	2.04 (0.4)	< 0.001
TC/HDL-C	4.65 (2.06)	4.19 (1.43)	3.98 (0.64)	0.052
TAG/HDL-C	3.56 (2.26)	3.52 (1.74)	4.41 (3.85)	0.041
HOMA-IR	0.99 (0.76)	0.99 (0.86)	1.83 (0.75)	< 0.001
HOMA-β	87 (54.92)	64.85 (32.32)	25.4 (19.50)	< 0.001
HOMA-S	99.85 (93.12)	101.2 (97.85)	54.60 (20.75)	< 0.001
QUICKI	0.36 (0.05)	0.35 (0.06)	0.29 (0.01)	< 0.001

Data are shown as median (IQR). Kruskal–Wallis was performed. One way ANOVA test was performed for TC

*BMI* body mass index, *FBG* fasting blood sugar, *HbA1c* hemoglobin A1c, *TC* total cholesterol, *TAG* triacylglycerol, *HDL-C* HDL cholesterol, *LDL-C* LDL cholesterol, *HOMA-IR* homeostatic model assessment of insulin resistance, HOMA-β, *QUICKI* quantitative insulin sensitivity check index, triacylglycerol (TAG) and total cholesterol/HDL cholesterol (TC/HDL-C) ratio

As shown in Table 5, in crude logistic regression model (model 1), OR = 1.547 (95% CI 1.293–1.850, P < 0.001) was observed for T2DM prevalence. The OR of T2DM, when adjusted for age, sex and BMI (model 2), was still significant [1.544 (95% CI 1.277, 1.867, P < 0.001)]. In model 3, when adjusted for age, sex, BMI and lipid profile (TAG, HDL-C, LDL-C, LDL-C, HDL-C, cholesterol/HDL-C) that may affect asprosin concentrations, OR = 1.332 (95% CI 0.903–1.963, P = 0.148) was lower for the T2DM prevalence compared to the second model, and it was not statistically significant (Table 5).

**Table 5:** ORs and 95% CIs for T2DM risk according to serum asprosin levels

	OR (95% CI)	
	Control (reference)	T2DM
Model 1 P-value	1	1.547 (1.293–1.850) < 0.001
Model 2 P-value	1	1.544 (1.277–1.867) < 0.001
Model 3 P-value	1	1.332 (0.903–1.963) 0.148

The adjusted model for potential variables. Model 1 is the crude model. Model 2 is adjusted for age, BMI and, gender. Model 3 is based on model 2 but further adjusted for TAG, HDL-C, LDL-C, LDL-C/HDL-C and also cholesterol/HDL-C

## Discussion

To the best of our knowledge, this is the first study on the association between serum concentrations of asprosin and lipid ratio used to predict the risk of cardiovascular disease in patients with T2DM. Also, our study adds to the limited available evidence on the association of asprosin and T2DM status.

We observed serum concentrations of asprosin in patients with T2DM are significantly higher than healthy controls. There was a positive and significant correlation between asprosin and FBG, HbA1c, TAG, HOMA-IR and TC/HDL-C ratio in T2DM patients. Also, serum concentrations of asprosin in subjects with T2DM were negatively and significantly correlated with QUICKI, HOMA-β, and HOMA-S. We found that regardless the T2DM situation, subjects with higher BMI had higher concentrations of asprosin compared to individuals with lower BMI.

### Interpretation of results

In the present study, serum concentrations of asprosin in patients with T2DM were significantly higher than healthy controls. Also, while subjects were divided into tertiles, the number of T2DM patients increased in harmony with tertiles. These findings are in agreement with the results of Zhang et al. study [20]. In our study, the OR value for T2DM prevalence was 1.547 (95% CI 1.293–1.850), indicated that 1 ng/L increase in asprosin concentration was associated with 55% increase in T2DM prevalence. However, the OR calculated in our study was lower than that of Zhang’s study [1.547 (95% CI 1.293–1.850) vs. 3.278 (95% CI 1.053–10.200)]. This difference is likely to be related to the statistical analysis as we used the control group as the reference group to calculate OR, while in Zhang’s study, individuals with serum concentrations of asprosin in the T1 range were used as a baseline to calculate OR. The concentration of asprosin measured in this study is close to reported concentration by Zhang’s study but different than Wang et al. study [19, 20]. Factors that may affect asprosin concentration have not yet been identified. But it may be due to differences in diet and lifestyle [28, 29].

In our study, there was a significant negative correlation between asprosin and HOMA-β and QUICKI in T2DM patients and positive correlation with HOMA-IR. Similar results were reported by Zhang et al. [21].

There was a significant relationship between serum concentrations of asprosin and BMI in both T2DM patients and healthy controls. However, the relationship between asprosin and BMI was not clear. Zhang et al. reported that serum concentrations of asprosin were significantly correlated with adiposity-related parameters such as BMI, waist circumference and waist-hip ratio in T2DM, but in non-diabetic subjects, no significant relationship was found between asprosin and these parameters. However, in their study, there was a weak positive association between asprosin and BMI in the healthy group [20]. Wang et al. reported that asprosin had a significant weak correlation with WC (r = 0.185, P value = 0.027), however they did not find any significant correlation between asprosin concentration and WHR (r = 0.002, P value = 0.983), nor with BMI (r = 0.097, P value = 0.249) [19]. Therefore, further studies are needed to investigate the association between obesity-related parameters and serum concentrations of asprosin.

Our results showed that asprosin was significantly correlated with serum TAG concentration and TC/HDL-C ratio in T2DM patients, but there was no significant relationship between TAG concentration and TC/HDL-C ratio in healthy subjects. The TC/HDL-C ratio is used to predict the risk of ischemic heart disease [30]. According to prospective studies, increased TC/HDL-C ratio is associated with a higher risk of cardiovascular disease [31]. Regarding the extreme prevalence of the cardiovascular disease in patients with T2DM [32], further studies are recommended to determine the association between asprosin and the risk of cardiovascular disease development. Contrary to Zhang et al. study [20], we did not observe a relationship between asprosin and HDL-C concentrations in both groups.

### Biological plausibility

The association between asprosin and insulin resistance has been shown in previous studies [18, 20, 21]. Also, Lee et al. suggest the role of asprosin in the deterioration of pancreatic function as a fundamental pathophysiological mechanism in T2DM. They treated MIN6 cells and primary human islets with recombinant asprosin and showed that asprosin in a dose-dependent manner induced inflammatory response, cellular dysfunction, and apoptosis in these cells [33]. Moreover, changes in lipid metabolism may also be due to insulin resistance induction in the liver [34]. Several studies have shown that insulin modulates the metabolism of TAG by mechanisms such as activating or inactivating metabolic pathways enzymes or altering gene expression [34].

### Limitations

Finally, our study has several limitations. The sample size was relatively limited and the findings of this study required to confirm in other ethnicities. Our observational design had limited capability to address the underlying signaling pathways. We were unable to investigate the effect of other factors that may have an impact on asprosin secretion, such as diet and physical activity. Also, we used BMI as the only adiposity index to examine the relationship between the asprosin and obesity and the correlation with other indexes were not investigated.

## Conclusion

We found serum concentrations of asprosin were significantly higher in patients with newly diagnosed T2DM. The serum concentration of asprosin was positively correlated with FBG, HbA1c, TAG, insulin resistance indices (HOMA-IR) and CVDs risk factor (TC/HDL-C) and was negatively correlated with insulin sensitivity indices (HOMA-S, and QUICKI) in T2DM patients. Therefore, it seems asprosin is correlated with the metabolism of glucose and lipids and insulin resistance in patients with T2DM. Based on these results and findings of previous studies, asprosin could affect glucose and lipids metabolism; therefore, asprosin could have an important role in therapeutic or diagnostic goals for diabetes. We observed that although BMI was similar between the two groups, asprosin concentration was higher in patients with T2DM. This finding suggested that asprosin regulation is abnormal in patients with T2DM. However, further studies on other populations with larger sizes are recommended to confirm this hypothesis and other potential mechanisms.

## Data Availability

None.
